# QSAR analysis of immune recognition for triazine herbicides based on immunoassay data for polyclonal and monoclonal antibodies

**DOI:** 10.1371/journal.pone.0214879

**Published:** 2019-04-03

**Authors:** Andrey A. Buglak, Anatoly V. Zherdev, Hong-Tao Lei, Boris B. Dzantiev

**Affiliations:** 1 A. N. Bach Institute of Biochemistry, Research Center of Biotechnology of the Russian Academy of Sciences, Moscow, Russia; 2 St. Petersburg State University, St. Petersburg, Russia; 3 Guangdong Provincial Key Laboratory of Food Quality and Safety, South China Agricultural University, Guangzhou, China; Meiji Pharmaceutical University, JAPAN

## Abstract

A common task in the immunodetection of structurally close compounds is to analyze the selectivity of immune recognition; it is required to understand the regularities of immune recognition and to elucidate the basic structural elements which provide it. Triazines are compounds of particular interest for such research due to their high variability and the necessity of their monitoring to provide safety for agricultural products and foodstuffs. We evaluated the binding of 20 triazines with polyclonal (pAb) and monoclonal (mAb) antibodies obtained using atrazine as the immunogenic hapten. A total of over 3000 descriptors were used in the quantitative structure-activity relationship (QSAR) analysis of binding activities (pIC_50_). A comparison of the two enzyme immunoassay systems showed that the system with pAb is much easier to describe using 2D QSAR methodology, while the system with mAb can be described using the 3D QSAR CoMFA. Thus, for the 3D QSAR model of the polyclonal antibodies, the main statistical parameter q^2^ (‘leave-many-out’) is equal to 0.498, and for monoclonal antibodies, q^2^ is equal to 0.566. Obviously, in the case of pAb, we deal with several targets, while in the case of mAb the target is one, and therefore it is easier to describe it using specific fields of molecular interactions distributed in space.

## Introduction

Triazines are herbicides which are widely used in agriculture and may accumulate in soil, as well as in food products [1]. Triazines are bound in soil to solids as well as to dissolved fractions of humic and fulvic acids, which leads not only to the accumulation of triazines in soil but also to the contamination of surface and ground waters [2] since triazines are water soluble.

Triazines may undergo chemical transformations of both a biotic and abiotic nature. The parent compound atrazine is subjected to oxidation of the alkyl substituents, oxidative dealkylation, hydroxylation, and also to ring cleavage [3–5]. The derivatives of atrazine may be toxic to a greater or lesser extent. Triazines may induce different physiological disorders in humans and animals such as immunity suppression [6] and birth defects [7]. Thus, contamination of the environment by triazine herbicides is a significant risk factor. For this reason, it is necessary to monitor the triazine levels in soil, water, agricultural products and foods.

The methods of triazine detection, such as NMR, HPLC, mass-spectrometry and others, are time- and labour-consuming. The immunoassay determination of triazines is much easier, less expensive, highly sensitive and rapid. Due to its selectivity and simplicity, the immunoassay has been used to detect a wide variety of environmental pollutants including triazines [8–10]. However, the significant variety of triazines and their derivatives makes the detailed analysis of the regulations of their immune recognition necessary.

**Fig 1:**
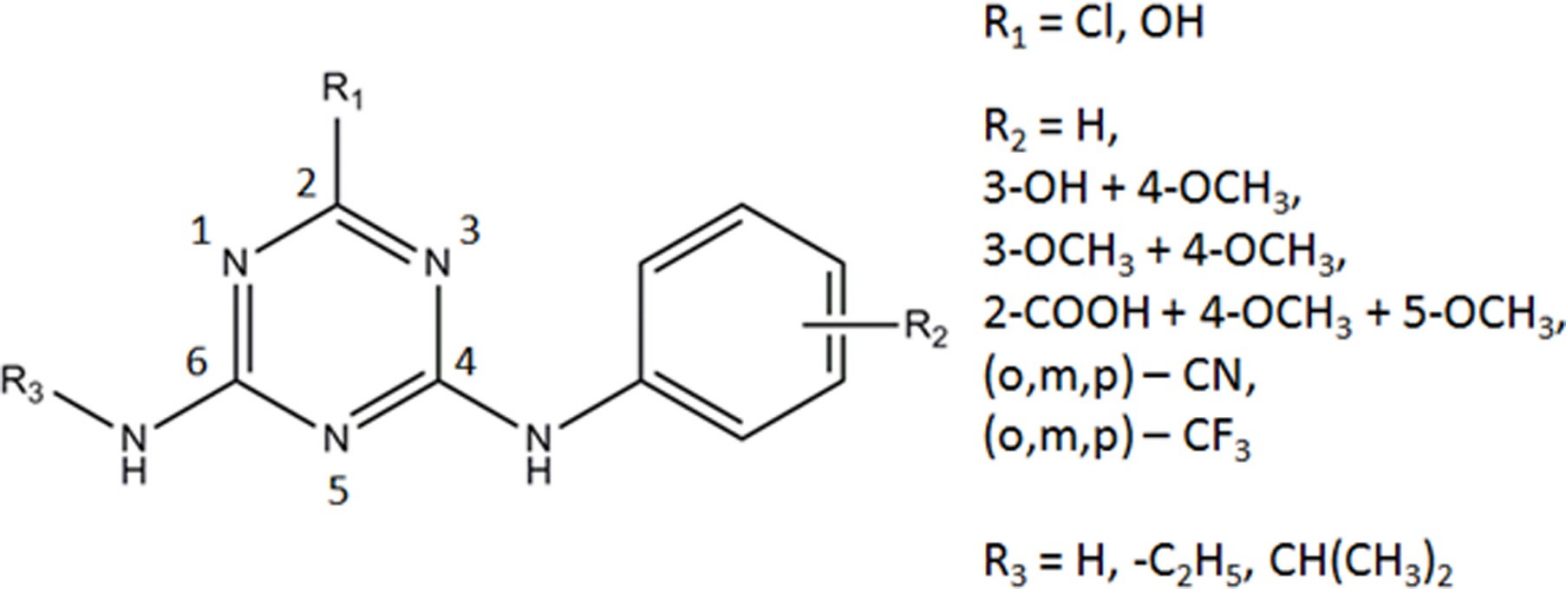
Molecular structure of arylamino-s-triazines (based on Dankwardt et al., 1996).

The quantitative structure-activity relationship (QSAR) is widely used to study the immune recognition of different classes of toxic food contaminants and veterinary drugs, including pesticides, etc. [11–14]. The work by Yuan and co-authors reported an immunoassay analysis of triazines; however, on only a small set of 11 compounds [9]. QSAR is used for the analysis of immunoassays on quinolones and fluoroquinolones [15–19], organophosphorus pesticides [20–21], phenylurea herbicides [22], and sulfonamides [23]. In this study, we used molecular modeling to study triazine recognition by monoclonal and polyclonal antibodies. Twenty triazines along with 2D and 3D-QSAR methodology were used to study the relationship between the antigen and antibody. The experimental data of microplate immunoenzyme assays with broad specificity for triazines were used from a classical work by A. Dankwardt and co-authors [24]. The comparative immunoassay of polyclonal and monoclonal antibodies used 4-arylamino-6-amino-1,3,5-triazines (Fig 1). Antibodies were grown using atrazine as an immunizing hapten.

The antibody-binding activity of triazines from Table 1, presented in the logarithmic form (pIC_50_ = -log IC_50_), was used as the dependent variable y during QSAR calculations. In Table 1, S2 stands for polyclonal sheep antibodies, while K4E7 stands for monoclonal antibodies.

**Table 1:** Cross-reactivity (CR) and concentration of 50% inhibition for the interaction of triazines with polyclonal (S2) and monoclonal antibodies (K4E7), according to Dankwardt et al., 1996.

№	Compound	CR, %		IC_50_, nmol l^-1^	
		S2	K4E7	S2	K4E7
1	2-chloro-4-isopropylamino-6-ethylamino-1,3,5-triazine (atrazine)	100	100	0.93	0.37
2	2-chloro-4-isopropylamino-6-amino-1,3,5-triazine (deethylatrazine)	15	18	6.0	2.1
3	2-chloro-4-ethylamino-6-amino-1,3,5-triazine (deisopropylatrazine)	0.4	1.8	232	20.6
4	2-hydroxy-4-isopropylamino-6-ethylamino-1,3,5-triazine (hydroxyatrazine)	6	0.1	15.3	371
5	2-chloro-4-anilino-6-isopropylamino-1,3,5-triazine	106	140	0.88	0.27
6	2-chloro-4-(3’,4’-dimethoxyanilino)-6-isopropylamino-1,3,5-triazine	126	140	0.74	0.27
7	2-chloro-4-(3’,4’-dimethoxybenzylamino)-6-isopropylamino-1,3,5-triazine	109	148	0.84	0.25
8	2-chloro-4-(3’-hydroxy-4’-methoxyanilino)-6-isopropylamino-1,3,5-triazine	74	79	1.25	0.47
9	2-chloro-4-(2’-carboxy-4’,5’-dimethoxyanilino)-6-isopropylamino-1,3,5-triazine	9	<0.1	10.3	>500
10	2-chloro-4-(2’-nitrilanilino)-6-isopropylamino-1,3,5-triazine	134	70	0.70	0.52
11	2-chloro-4-(3’-nitrilanilino)-6-isopropylamino-1,3,5-triazine	134	108	0.70	0.34
12	2-chloro-4-(4’-nitrilanilino)-6-isopropylamino-1,3,5-triazine	101	107	0.93	0.35
13	2-chloro-4-(2’-triflourmethylanilino)-6-isopropylamino-1,3,5-triazine	91	81	1.02	0.46
14	2-chloro-4-(3’-triflourmethylanilino)-6-isopropylamino-1,3,5-triazine	133	75	0.70	0.46
15	2-chloro-4-(4’-triflourmethylanilino)-6-isopropylamino-1,3,5-triazine	92	129	1.02	0.49
16	2-chloro-4-(3’,4’-dimethoxyanilino)-6-ethylamino-1,3,5-triazine	5	10	18.6	3.68
17	2-hydroxy-4-anilino-6-isopropylamino-1,3,5-triazine	0.4	0.2	232	186
18	2-hydroxy-4-(3’,4’-dimethoxyanilino)-6-isopropylamino-1,3,5-triazine	0.4	0.2	232	186
19	2-chloro-4-anilino-6-amino-1,3,5-triazine	0.1	<0.1	500	>500
20	2-chloro-4-(3’,4’-dimethoxyanilino)-6-amino-1,3,5-triazine	<0.1	<0.1	>500	>500

## Materials and methods

### Conformational analysis and geometry optimization

The preparation of molecular geometries for 2D and 3D QSAR analysis was carried out in the Spartan v.16 program. A series of conformers for each of the 20 compounds was obtained by the systematic search method using the MMFF force field [25]. The optimization of the geometry for each conformer was carried out using the semi-empirical AM1 method [26]. In order to confirm the correspondence between the obtained geometry and the minimum surface of the potential energy for each conformer, the Hessian was calculated. The conformers with the lowest total energy were used for 2D QSAR analysis.

### 2D QSAR

Using a random number generator, triazines were divided into two samples: the training set (80%) and test set (20%). The requirements for the maximum and minimum values in the test sample were as follows: 1) the maximum value of pIC_50_ should be less than or equal to the maximum value of pIC_50_ in the training sample; and 2) the minimum value of pIC_50_ must be greater than or equal to the minimum value of pIC_50_ in the training sample. The actual limit IC_50_ values given in Table 1 are equal to 500 nmol l^-1^, which were experimentally tested values in the original work by Dankwardt et al., 1996 [24]. When the activity IC_50_ was equal to or greater than 500 nmol l^-1^ the pIC_50_ value of 6.30103 was used.

The required linear regression equation should have the following form:

y=a1*x1+…an*xn+c

where *y* is the dependent variable (pIC_50_); *a*_*1*_ and *a*_*n*_ are regression coefficients; *x*_*1*_ and *x*_*n*_ are independent variables (descriptors); and *c* is a regression constant.

We estimated the contribution of a descriptor to the model using the following equation:

$$\alpha \left(x_{1}\right)=\frac{R^{2}\left(x_{1},x_{2},x_{3}\right)-R^{2}\left(x_{2},x_{3}\right)}{3\times R^{2}\left(x_{1},x_{2},x_{3}\right)-R^{2}\left(x_{1},x_{2}\right)-R^{2}\left(x_{1},x_{3}\right)-R^{2}\left(x_{2},x_{3}\right)}\times 100\%$$

where *α(x*_*1*_*)* is the relative contribution of the descriptor *x*_*1*_ to the model with three descriptors; $R^{2}\left({x_{1},x}_{2},x_{3}\right)$ is the determination coefficient of the model with all three descriptors; and $R^{2}\left(x_{2},x_{3}\right)$ is the determination coefficient of the model with two descriptors: *x*_*2*_ and *x*_*3*_.

During the 2D QSAR analysis, different types of descriptors were used: constitutional (number of hydrogen bond donors, number of hydrogen bond acceptors, number of rings, number of chains, number of CH_3_ groups, number of OH groups, etc.), electrostatic descriptors (maximum positive charge, maximum negative charge, the number of positively charged atoms, the number of negatively charged atoms, etc.), topological descriptors (kappa-indices describing the shape of the molecule, the indices of molecular bonds of Kier and Hall, etc.), 3D descriptors (volume, surface area, length and area of the projection on the coordinate axis, etc.), physico-chemical (lipophilicity (LogP), molecular refraction, polarizability, solubility in water, etc.) and quantum-chemical descriptors (energy of frontier molecular orbitals, electrostatic charges of atoms, the population of atoms according to Mulliken, etc.), amounting to a total of more than 3000 descriptors. The values of the descriptors were calculated using the program packages Spartan v. 16 and E-Dragon 1.0.

For the QSAR obtained model, the cross-validation was performed. Also, the models were tested for their predictive power using an external test set of compounds. The following statistical indicators were used:

- r^2^—coefficient of determination for the training sample [27];
- The determination coefficient R^2^adj, corrected for the number of descriptors involved in the model;
- q^2^—r^2^ from the results of the internal cross-validation of the training sample using the leave-one-out (LOO) method [28,29];
- LOF—error of Friedman approximation (Friedman Lack of Fit);
- RMSE—root-mean-square error;
- Max Error—the maximum prediction error for all the compounds in the test and training set;
- pred_r^2^—r^2^, which estimates the predictive ability of the model relative to the test set.

### 3D QSAR

#### Spatial alignment of molecules

**Fig 2:**
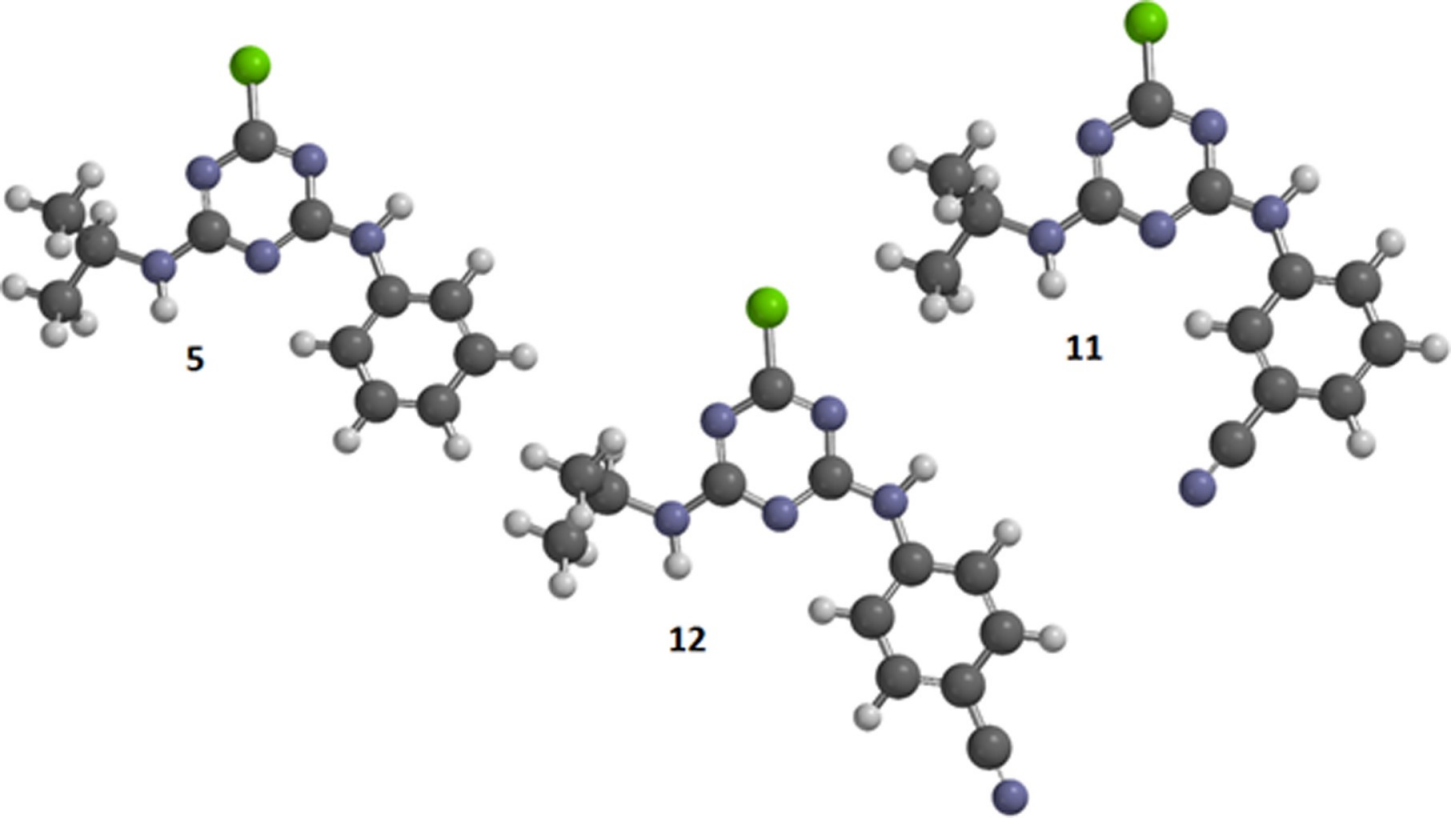
The most energetically favorable conformations of compounds 5, 11 and 12 according to the calculation by HF/6-31G(d) method.

The alignment of molecules plays a key role in 3D-QSAR. As a rule, the most energetically favorable conformation of the most active compound or the geometry corresponding directly to the interaction of the ligand and target (antibody), obtained on the basis of X-ray diffraction analysis or docking data, is chosen as the template for the alignment [30]. In our case, the most active conformation is unknown, and a number of the most active compounds (**10**, **14**) have a large number of conformers, which makes it difficult to analyze them correctly using quantum-chemical methods of required accuracy. Compounds **5**, **11** and **12** are also highly active, but with a small number of conformers compared to other triazines in the sample. All conformers of compounds **5** (12 conformers), **11** (23 conformers) and **12** (12 conformers) were optimized using the Hartree–Fock method and 6-31G(d) basis set (Fig 2). It was found that the most energetically favorable conformers of these compounds are identical, and therefore it can be assumed that this particular conformation is the most active and corresponds to the interaction with the antibody.

**Fig 3:**
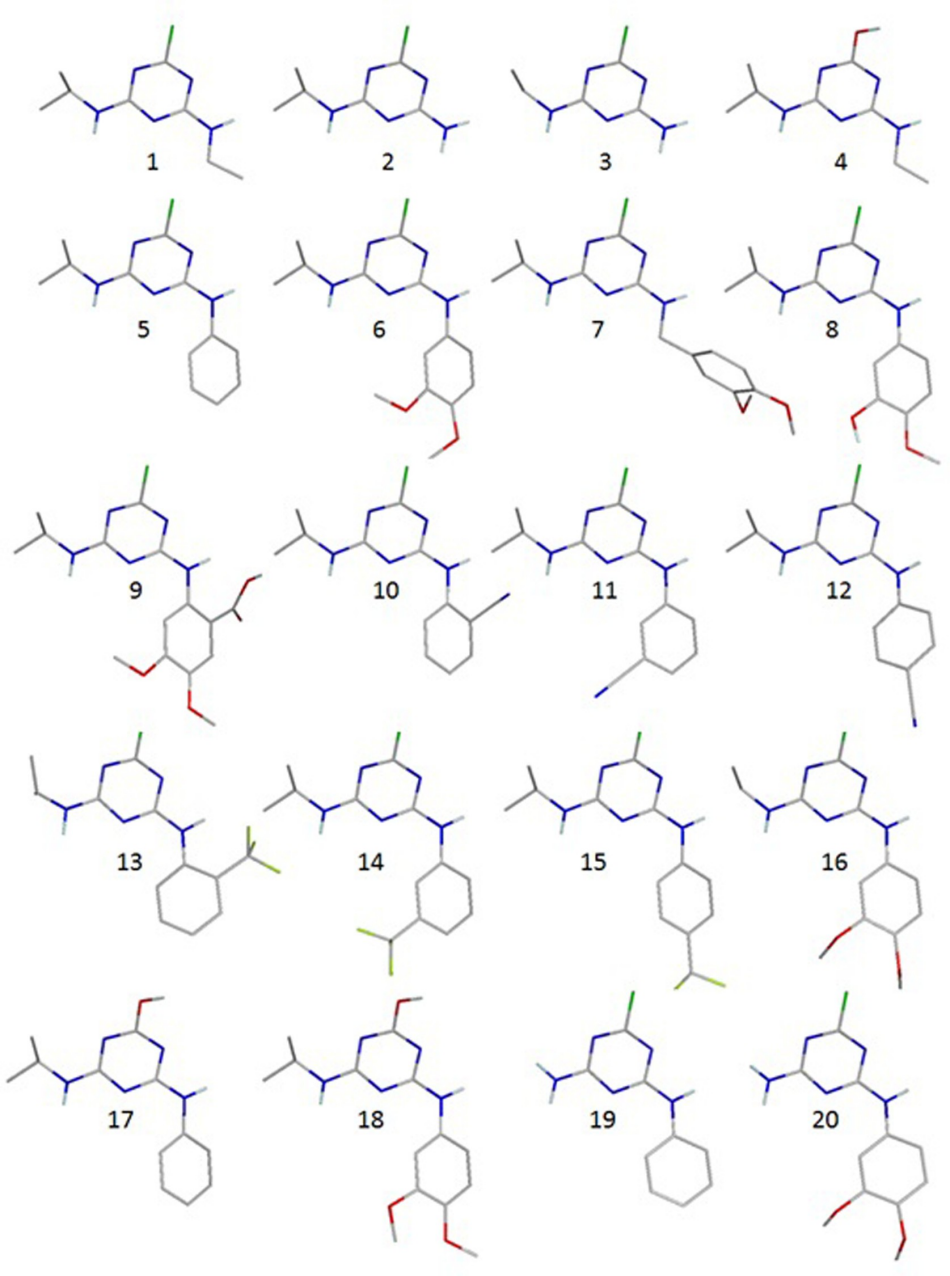
Triazine geometries optimized with the AM1 method (hydrogen atoms are represented only in -OH groups).

Triazine molecules in various conformations, optimized with the AM1 method, were superimposed on the template (compounds **5** and/or **11**) using the algorithm presented in the open3DALIGN program [31]. Conformations corresponding to the largest overlap with the atoms of compounds **5** and **11** were selected (Fig 3).

The aligned geometries (S1 Fig) were used to create and analyze the fields of molecular interactions using the open3DQSAR program [32]. Visualization of 3D structures and contour maps of the force fields was carried out in the PyMol program.

#### Building of molecular interaction fields (MIF)

Molecular interaction fields (MIF) were calculated to analyze the pIC_50_ values of triazines and to create CoMFA models for the S2 and K4E7 systems.

Two potentials were used to create the fields of molecular interaction: the steric potential in the form of the Lennard–Jones function 6–12 between the atoms of the molecule and the sp3 carbon atom:

$${\text{E}}_{\text{VdW}}=\sum_{\text{i}=1}^{\text{n}}\left[\frac{{\text{A}}_{\text{i}}}{{\text{r}}_{\text{i}}^{12}}-\frac{{\text{B}}_{\text{i}}}{{\text{r}}_{\text{i}}^{6}}\right];$$

The electrostatic field was calculated by summing the Coulomb interactions between the test atom with charge +1 and triazine molecules:

$$E_{ele}=k\overset{n}{\underset{i=1}{\sum }}\left[\frac{q_{i}}{r_{i}^{m}}\right]$$

#### MIF preprocessing

The force-field data was preliminarily processed to remove non-informative *x* variables:

1. The values of the variables less than 0.05 kcal mol^-1^ were equated to zero;
2. Cut-off values were above 30 kcal mol-1 and below -30 kcal mol^-1^;
3. *x* variables with a standard deviation value below <0.1 were deleted;
4. Variables that had four or fewer nonzero values within the sample were removed.

#### Building of regression models

After all the preliminary operations were completed, we proceeded to construct the models using the partial least squares (PLS) method [33], based on the NIPALS algorithm (non-linear iterative partial least squares) [34]. The predictive power of the models was evaluated using the “leave-one-out” (LOO) cross-validation method:

$$q^{2}=1-\frac{\sum {\left(y_{obs}-y_{pred}\right)}^{2}}{\sum {\left(y_{obs}-y_{mean}\right)}^{2}};$$

Also, we used the “leave-many-out” cross-validation method: a sample of 20 compounds was randomly broken into the training (75%) and test (25%) parts. The q^2^ parameter was calculated for the test part. The partition procedure was repeated 50 times, and the average of the 50 partitioning variants was given the value q^2^ (LMO).

To estimate the internal stability and predictive power of the 3D models, the standard prediction error (SDEP) parameter was calculated:

$$SDEP=\sqrt{\sum \frac{{\left(y_{obs}-y_{pred}\right)}^{2}}{N}};$$

where $y_{obs}$ is the experimental value; $y_{pred}$ is the predicted value; $y_{mean}$ is the mean value; and *N* is the number of molecules in the sample.

Procedures for selecting variables and methods for creating clusters of variables with common characteristics were used in order to increase the predictive ability of models: the SRD (smart region definition) method [35] groups variables based on their localization in three-dimensional space. This procedure reduces the number of descriptors bearing the same information; and fractional factorial design (FFD) [36,37] allows the selection of variables that have the greatest impact on the predictive ability of models. The FFD selection was conducted based on the leave-many-out cross analysis, using the SRD of the variable group.

## Results

### 2D QSAR

#### S2 system analysis (polyclonal antibodies)

For the S2 system, a series of multiple linear regression models was obtained:

Model 1

pIC50=23.68+0.985*ES_Count_sssCH−64.66*Mulliken_Charge_C4+0.056*Solvation_E

Model 2

pIC50=12.43+4.393*ES_Sum_sssCH−107.3*Jurs_FPSA_3

Model 3

pIC50=11.92−0.910*CHI_2+1.932*CHI_V_2−100.3*Jurs_FPSA_3

Model 4

pIC50=8.807+5.366*ES_Sum_sssCH+0.828*IC2–104.0*Jurs_FPSA_3

Model 5

pIC50=6.302+6.182*ES_Sum_sssCH+0.946*IC2+0.062*Solvation_E

It is believed that QSAR is predictive if the following conditions are satisfied: R^2^ > 0.6, q^2^ > 0.5 and pred_R^2^ > 0.5. Table 2 shows that all the presented models are statistically reliable and possess a predictive ability. Model 5 has the highest statistical parameters among the five models.

**Table 2:** Statistical parameters of the models for the S2 system.

№	Statistical parameter	Model 1	Model 2	Model 3	Model 4	Model 5
1	*R*^*2*^	0.941	0.890	0.937	0.937	**0.945**
2	*R*^*2*^ *adj*	0.926	0.873	0.921	0.921	**0.932**
3	*q*^*2*^	0.893	0.843	0.898	0.873	**0.900**
4	*pred_R*^*2*^	0.913	0.924	0.870	0.920	**0.942**
5	*Max error*	0.567	0.904	0.746	0.549	**0.454**
6	*RMS error*	0.295	0.387	0.305	0.306	**0.284**
7	*LOF*	0.383	**0.329**	0.411	0.412	0.355

Let us consider the descriptors involved in Model 5. *Solvation_E* (relative contribution, *α* = 66.8%) is the solvation energy calculated with the AM1 method. Compound **18** has the highest solvation energy value (-20.27 kJ mol^-1^), while compound **13** has the lowest (-71.22 kJ mol^-1^) (S1 Table). The lower the solvation energy, the higher the solubility. However, the energy of solvation has a negative value, and therefore compounds that are less soluble in water have higher *pIC*_*50*_ values.

*ES_Sum_sssCH* (26.1%) is the electrotopological index of methantriyl groups [38]. The *ES_Sum_sssCH* value is directly proportional to the activity of the molecule (**1**, **2** and similar compounds). *ES_Sum_sssCH* is equal to zero for compounds without >CH-groups. Obviously, the presence of the *ES_Sum_sssCH* descriptor in Model 5 indicates the participation of >CH-groups of triazine compounds in van der Waals interactions with antibodies.

*IC2* (7.1%) is the information content index (neighborhood symmetry of 2-order). This descriptor makes it possible to estimate the degree of heterogeneity of the molecular structure. Compound **1** has the lowest value of *IC2 (*3.431*)*, and compound **9** has, on the contrary, the highest value of this descriptor (4.426). *IC2* can be considered as a measure of the complexity of the triazine topology. *IC2* makes a minor contribution to the model (α = 7.1%).

**Fig 4:**
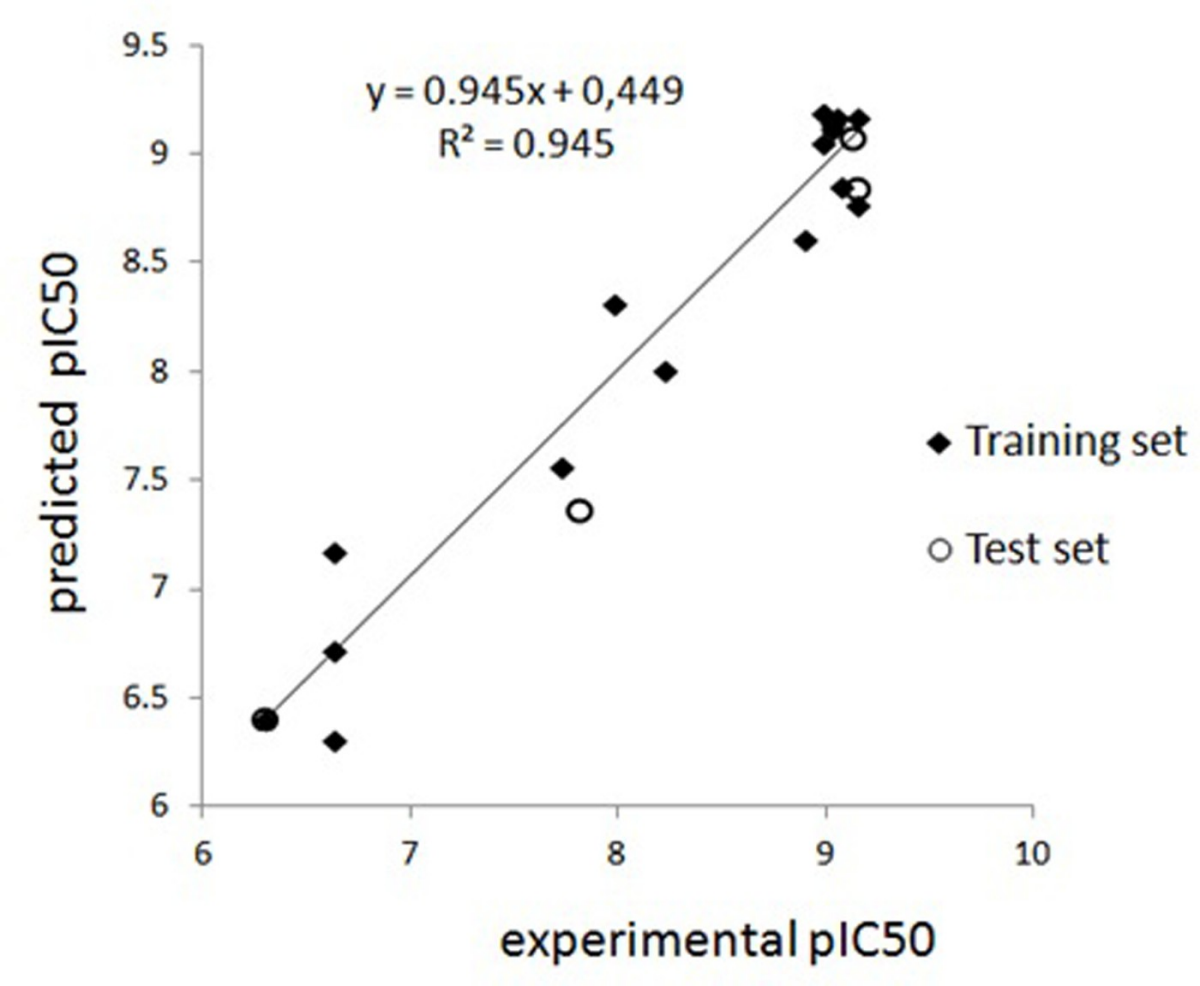
Comparison of experimental pIC_50_ values for triazine interactions with polyclonal antibodies (S2) and predicted activity based on Model 5; compounds 4, 6, 14 and 19 were used as a test set.

Experimental and predicted pIC_50_ values for triazine interactions with polyclonal antibodies (S2) based on Model 5 are presented on Fig 4.

#### K4E7 system analysis (monoclonal antibodies)

For the K4E7 system, a series of multiple linear regression models was obtained:

Model 6

pIC50=7.142−9.584*Mor21v−0.561*NPlusO_Count+33.83*R6m+

Model 7

pIC50=5.859−0.018*Jurs_TPSA−7.118*Mor21v+27.20*R6m+

Model 8

pIC50=−3.941+1.291*ES_Count_sssCH+5.271*IC2−0.216*Jurs_DPSA_3

Model 9

pIC50=7.173−8.061*Mor21v−0.0385*TPSA_NO+34.56*R6m+

Model 10

pIC50=7.369+1.637*ES_Count_sssCH+0.287*ES_Sum_sCl−0.791*Num_H_Donors_Lipinski

Model 10 has a high predictive ability, and its statistical parameters are higher than for models 6–9 (Table 3).

**Table 3:** Statistical parameters of 2D QSAR models for the K4E7 system.

№	Statistical parameters	Model 6	Model 7	Model 8	Model 9	Model 10
1	*R*^*2*^	0.921	0.915	0.917	0.925	**0.966**
2	*R*^*2*^ *adj*	0.902	0.894	0.896	0.906	**0.957**
3	*q*^*2*^	0.819	0.778	0.862	0.832	**0.939**
4	*pred_R*^*2*^	0.767	0.788	0.898	0.704	**0.969**
5	*Max error*	0.947	1.110	1.065	0.913	**0.469**
6	*RMS error*	0.431	0.449	0.444	0.422	**0.285**
7	*LOF*	0.822	0.890	0.869	0.787	**0.359**

*Num_H_Donors_Lipinski* (*α* = -37.9%) is the number of hydrogen bond donors (S2 Table). The value of the descriptor is inversely correlated with the activity: the greater the number of hydrogen bond donors, the lower the activity. Apparently, the presence of amine groups (-NH- and -NH2) negatively affects the activity.

*ES_Sum_sCl* (34.6%) is the electrotopological index of chlorine atoms.^35^ The *ES_Sum_sCl* value is directly proportional to the activity of the molecule. Apparently, the chlorine atom, as an electron-acceptor substituent, participates in electrostatic interactions.

*ES_Count_sssCH* (27.4%) is the number of methantriyl groups. Obviously, the presence of the *ES_Count_sssCH* descriptor in Model 10 indicates the participation of bulky substituents of triazine compounds in steric interactions with antibodies.

**Fig 5:**
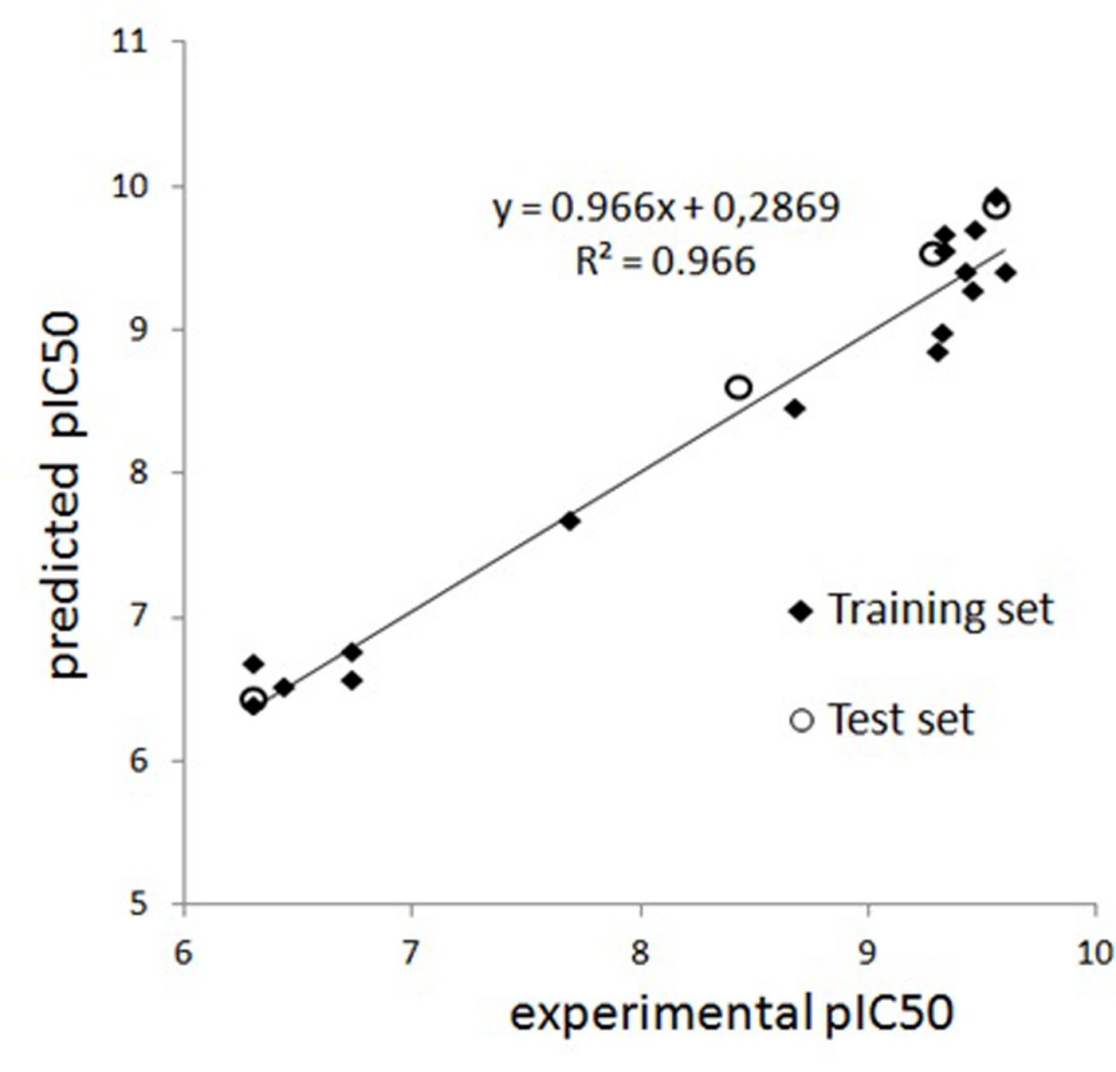
Comparison of experimental pIC_50_ values for triazine interactions with monoclonal antibodies (K4E7) and predicted activity based on Model 10; compounds 6, 10, 16 and 20 were used as a test set.

The experimental and predicted pIC_50_ values for triazine interactions with monoclonal antibodies (K4E7) based on Model 10 are presented in Fig 5.

### 3D QSAR

#### S2 system study

A 3D QSAR analysis was carried out for all 20 compounds. The interaction energy of the "probe" (carbon atom with charge +1) with the target molecule was calculated at each point of the regular 3D lattice. The dimensions of the 3D cubic grid were 26 × 18 × 22 Å. The lattice spacing was 2 Å.

One can consider Table 4 to compare the accuracy of the activity prediction for individual molecules within the model.

**Table 4:** Experimental and predicted values of the triazine cross-reactivity logarithm (pIC_50_) in the S2 system.

№	Experiment	Prediction
1	9.032	9.056
2	8.222	8.405
3	6.635	6.846
4	7.815	7.297
5	9.056	8.641
6	9.131	8.763
7	9.076	9.393
8	8.903	8.713
9	7.987	7.878
10	9.155	9.492
11	9.155	9.276
12	9.032	8.811
13	8.991	9.147
14	9.155	9.108
15	8.991	9.016
16	7.731	7.406
17	6.635	7.039
18	6.635	6.950
19	6.301	6.529
20	6.301	6.170

Van der Waals interactions make the main contribution to the interactions between the triazine and antibody in the S2 system in all three models (S3 Table).

To estimate the predictive ability of the 3D QSAR models, the following statistical parameters were used: the coefficient of determination (R^2^), the mean-square error of the model (SDEC), the F-statistic value, the correlation coefficient of the leave-one-out (LOO) method (q^2^_(LOO)_), the correlation coefficient of the LMO method (q^2^_(LMO)_), and the mean-square error of prediction (SDEP). The S2 system 3D QSAE model showed satisfactory statistical results (Table 5).

**Table 5:** Statistical parameters of the CoMFA model for the S2 system. SDEP: mean-square error of prediction. LOO: leave-one-out.

№	R^2^	SDEC	F-test	q^2^_(LOO)_	SDEP_(LOO)_	q^2^_(LMO)_	SDEP_(LMO)_
3	0.938	0.269	80.39	0.680	0.610	0.498	0.754

**Fig 6:**
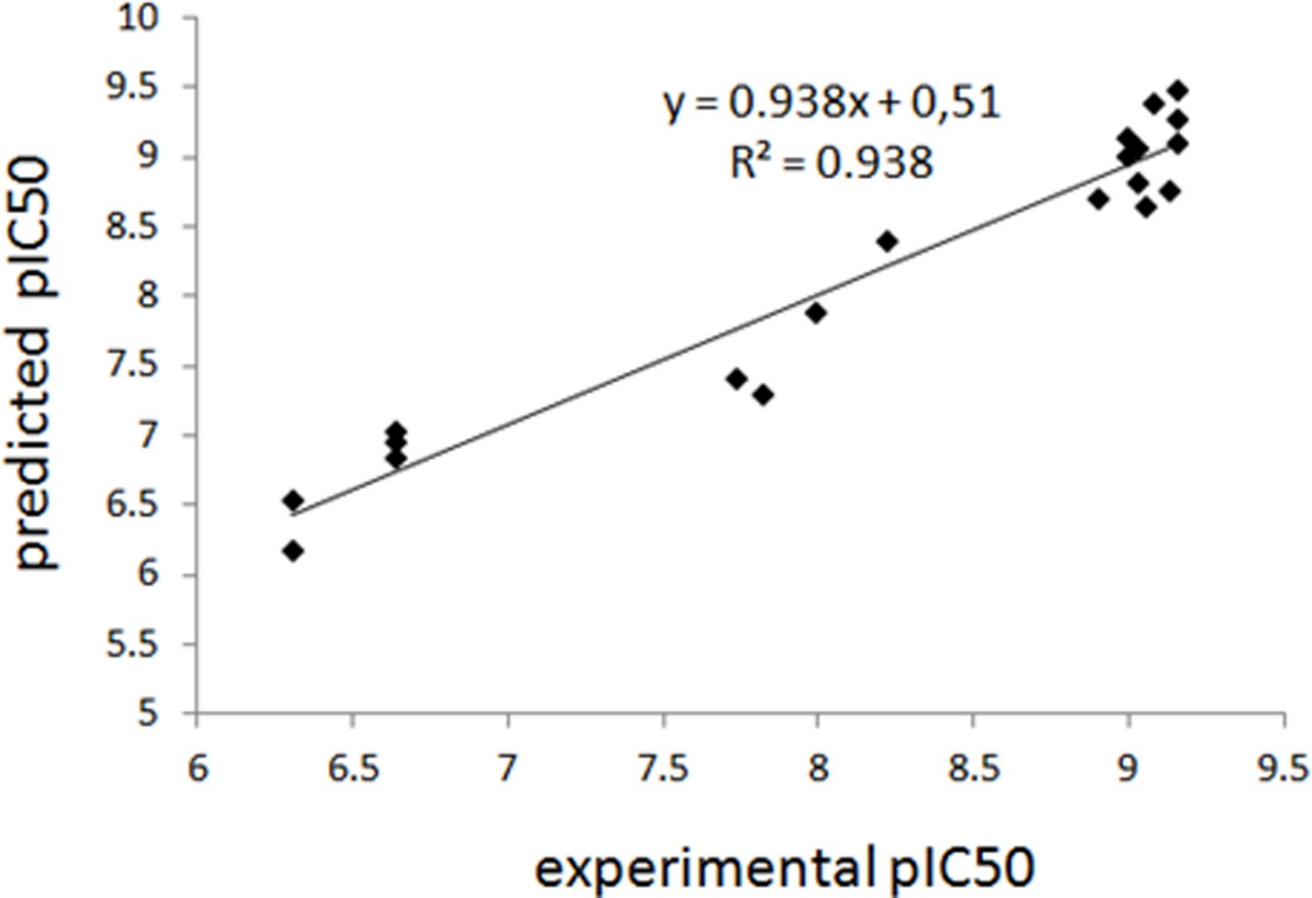
Experimental vs. predicted values of triazine cross-reactivity in the S2 system according to the CoMFA method (3D QSAR).

As can be seen from Table 5, the model has high predictive ability. High coefficients of determination (R2 = 0.938) and LOO cross-validation (q^2^ = 0.68) indicate the statistical significance of the obtained model. The high correlation between predicted and experimental values of the model can be seen in Fig 6.

**Fig 7:**
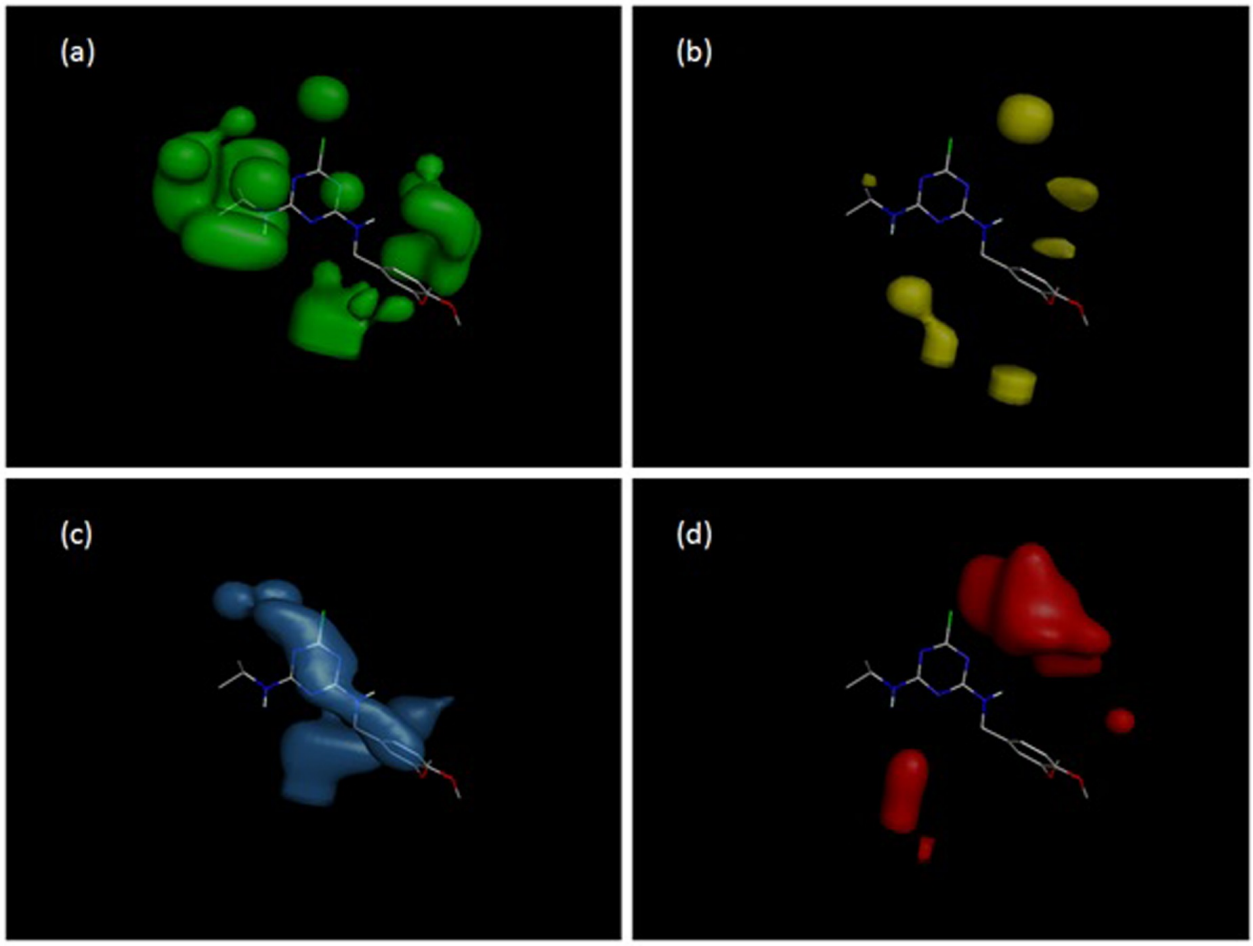
The molecular interaction field (MIF) contour maps for system S2 and compound **7**: (a) favorable steric interactions; (b) unfavorable steric interactions; (c) sites favorable for positively charged groups; (d) sites favorable for negatively charged groups.

Molecular interaction field (MIF) contour maps were obtained to visualize the information about the 3D-QSAR models. Contour maps are presented in Fig 7. The steric fields presented by green contours are favorable for bulk substituents and have a positive effect on cross-reactivity (Fig 7A), while the yellow contours represent the areas where the presence of bulk substituents is unfavorable for high activity (Fig 7B). The electrostatic fields are represented by blue and red contours. The blue contour reflects the areas where positively charged atoms have a positive effect on activity (Fig 7C), while the red contours reflect the regions in which the presence of negatively charged atoms is favorable for high activity (Fig 7D).

#### K4E7 system study

A 3D QSAR analysis was carried out for all 20 compounds without splitting the sample into the training and test parts. The same alignment was used as for the S2 system.

**Fig 8:**
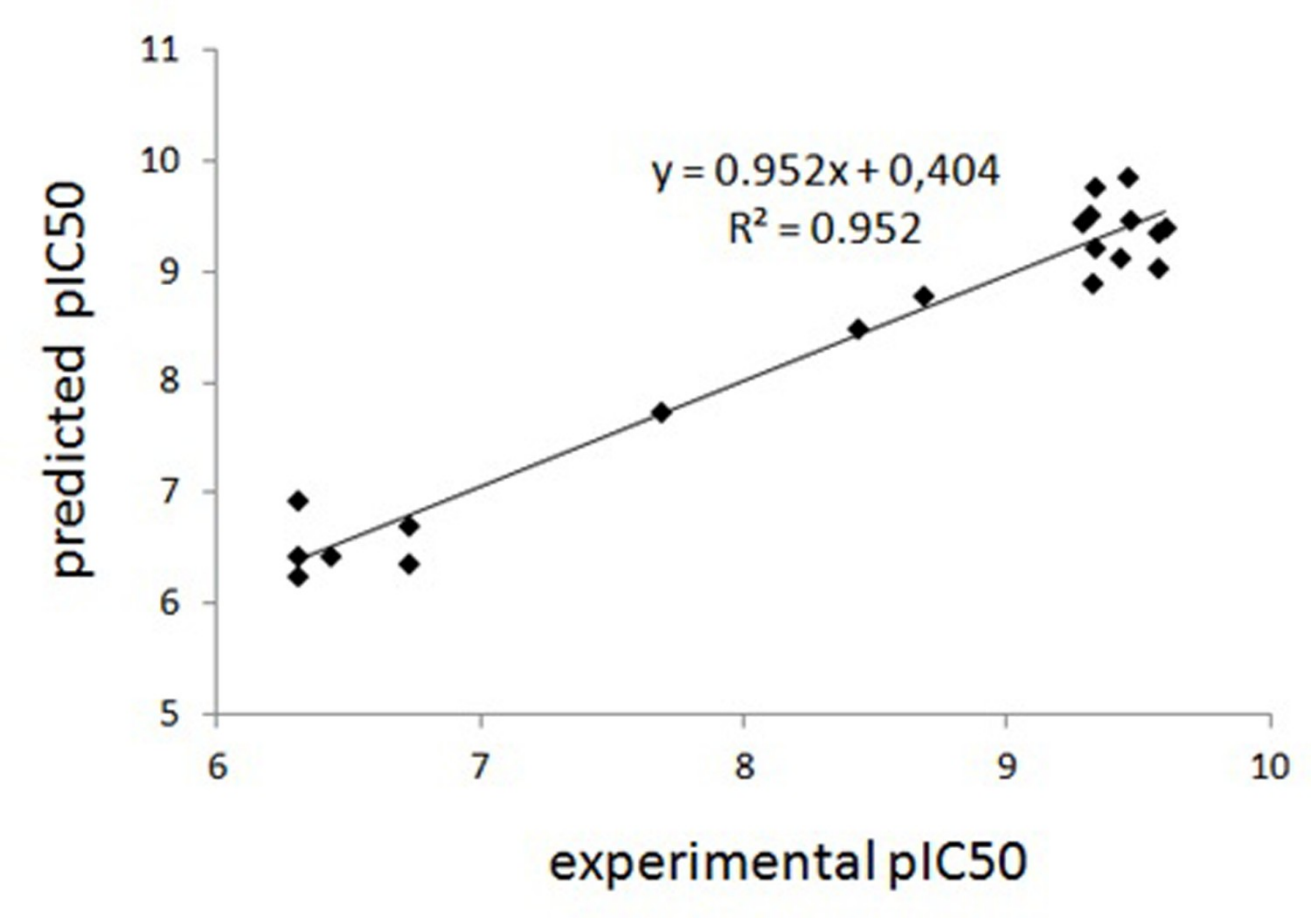
K4E7 system’s experimental and predicted pIC_50_ triazine values based on the CoMFA method (3D QSAR).

The predicted values of triazine activity are shown in Table 6. The calculated values of the model have the best match with the experimental data. The level of correlation between the activities predicted by the CoMFA model and the experimental values is also shown in Fig 8.

**Table 6:** Experimental and predicted values of triazine cross-reactivity in the K4E7 system.

№	Experiment	Prediction
1	9.432	9.120
2	8.678	8.786
3	7.686	7.746
4	6.431	6.429
5	9.569	9.031
6	9.569	9.361
7	9.602	9.397
8	9.328	8.894
9	6.301	6.442
10	9.284	9.455
11	9.469	9.462
12	9.456	9.871
13	9.337	9.212
14	9.337	9.776
15	9.310	9.527
16	8.434	8.497
17	6.731	6.721
18	6.731	6.362
19	6.301	6.931
20	6.301	6.266

**Fig 9:**
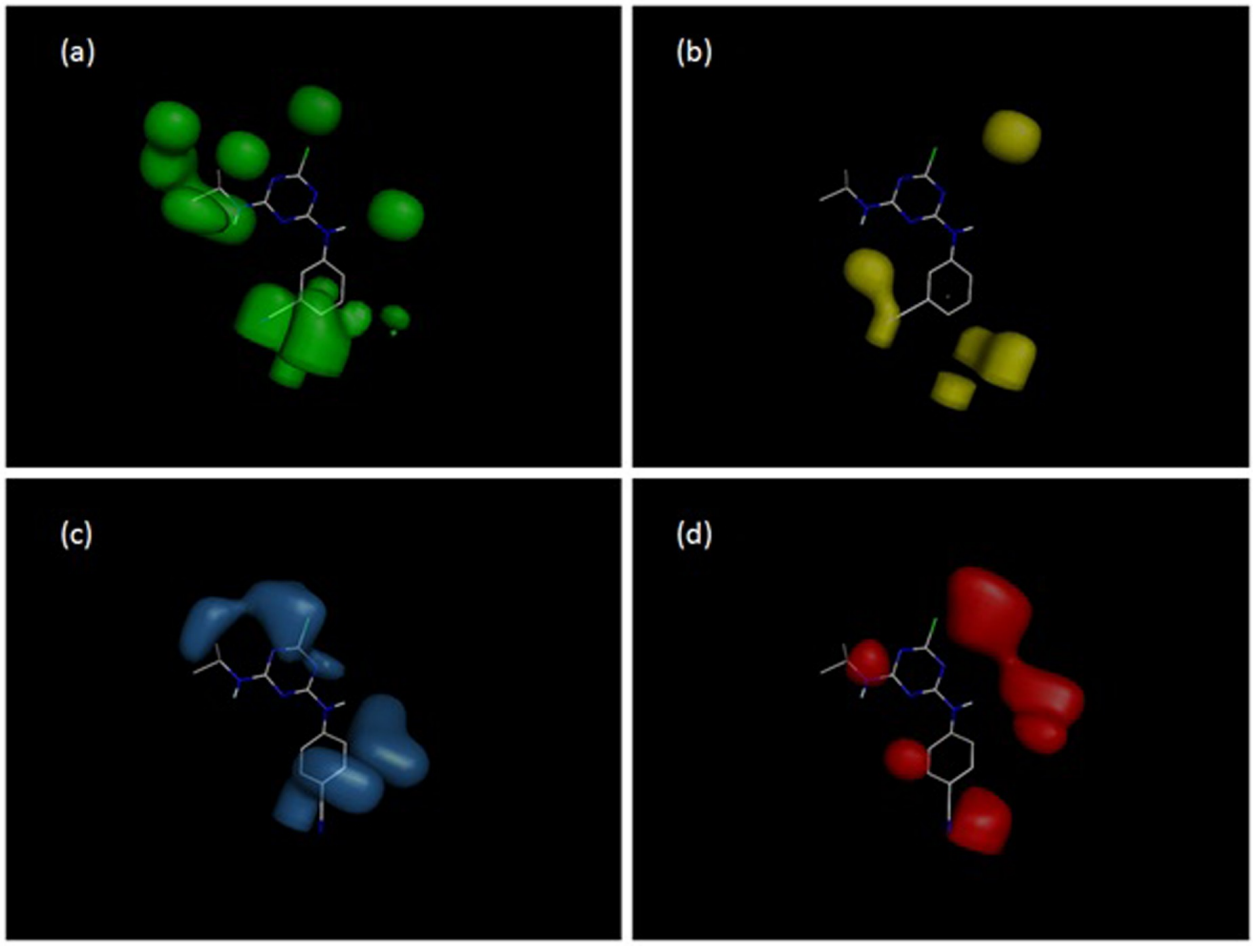
MIF contour maps for the K4E7 system: (a) favorable steric interactions and (b) unfavorable steric interactions for compound **11**; (c) sites favorable for positively charged groups; (d) sites favorable for negatively charged groups for compound **12**.

Table 7 shows that the model has high values of the determination coefficient (R^2^ = 0.952) and LOO cross-validation parameter (q^2^ = 0.637), which indicates the statistical significance of the model obtained. The contour maps of the molecular interaction for this model are presented in Fig 9.

**Table 7:** Statistical parameters of the 3D QSAR model for the K4E7 system.

№	R^2^	SDEC	F-test	q^2^_(LOO)_	SDEP_(LOO)_	q^2^_(LMO)_	SDEP_(LMO)_
3	0.952	0.291	105.0	0.637	0.796	0.566	0.861

The contributions of the van der Waals and electrostatic interactions to the model are approximately equal at 51% and 49%, respectively (S4 Table).

## Discussion

It seems reasonable to compare the most similar compounds with each other and find out which descriptors play a key role in their recognition.

Atrazine in the S2 system (polyclonal antibodies) exhibits significantly higher activity (CR 100%) than its metabolites: compounds **2**, **3** and **4** (CR 15%, 0.4% and 6%, respectively). Atrazine has the highest value of the *Solvation_E* descriptor (see S1 Table). The value of the *Solvation_E* descriptor is directly proportional to pIC_50_. The lower the *Solvation_E*, the higher the polarity of the molecule. For compounds 1–4, *Solvation_E* is equal to -33.49 kJ mol^-1^, -49.34 kJ mol^-1^, -56.07 kJ mol^-1^ and -58 kJ mol^-1^, respectively. In general, this is consistent with our observation that the polarity of triazines is unfavorable for the interaction with the antibodies. Also, compounds with a CR higher than 1% (compounds **1**, **2** and **4**) possess *ES_Sum_sssCH* with a non-zero value, while the compound with the lowest *ES_Sum_sssCH* value (compound **3**) has the lowest CR value– 0.4%.

For the K4E7 system (monoclonal antibodies), the highest pIC_50_ value of compound **1** among compounds **1**–**4** is determined by non-zero values of its descriptors *ES_Count_sssCH* and *ES_Sum_sCl*. Also, compound **1** has the lowest value of the descriptor *Num_H_Donors_Lipinski*. The descriptor *Num_H_Donors_Lipinski* is inversely correlated with pIC_50_.

For system S2, the highest CR value of compound **6** among compounds **6–9** (CR 126%, 109%, 74% and 9%, respectively) is determined by its lowest value of *E_Solvation* (-37.49 kJ mol^-1^), while *E_Solvation* is inversely correlated with CR.

In the case of the K4E7 system and compounds **6–9**, we should regard the CoMFA contour maps. The substituent at the C6 position does not differ in these compounds, and the variation of the substituent in the C4 position obviously plays the main role. The geometry of compound **7** is the most advantageous for the interaction with the antibody, and the geometry of **9** is the least advantageous (Table 1). The contour map of electrostatic charges (Fig 9D) indicates that the presence of a negatively charged carboxyl group at position 2' (compound **9**) is unfavorable for the binding with the antibody. Fig 9C, on the contrary, shows how the presence of negatively charged oxygen atoms of the methoxy groups of compound **7** has a positive effect on the activity. The contour map on Fig 9A shows that the aniline residue at the C4 position (namely, the benzene ring) generally has a positive effect on the activity of atrazines. Fig 9B shows that the bulky substituents localized at the meta position of the aniline have a negative effect on the activity; the methoxy substituents of compound 9 are located in these areas.

Compounds **10**, **11** and **12** have ortho-, meta- and para-positions of the nitrile of the aniline substituent, respectively (Fig 1). The contour maps of electrostatic interactions (Fig 7C and 7D) may explain why the activity of these compounds in system S2 is different. Fig 7D shows that the contour map is favorable for the interaction with the nitrile located at the 2' and 3' positions of the aniline ring.

Let us regard compounds 13, 14 and 15, which possess trifluoromethyl substituent (-CF3) in the ortho-, para- and meta- positions, respectively. In the S2 system, molecule 14 is more active than 13 and 15 (Table 1). In order to answer the question why these compounds exhibit different activities, it is required to consider the *Solvation_E* descriptor involved in 2D QSAR model 5. This descriptor is inversely correlated with pIC_50_. For compounds **13**, **14** and **15**, *Solvation_E* is equal to -20.27 kJ mol^-1^, -30.06 kJ mol^-1^ and -27.35 kJ mol^-1^, respectively. Obviously, the lower the solvation energy, the lower the activity. Also, the pIC_50_ of compounds **13**, **14** and **15** is correlated with *IC2* values of 4.15, 4.21 and 4.09, respectively. The higher value of *IC2* descriptor for compound **14** means a higher heterogeneity of **14** among compounds **13–15**.

Obviously, the low activity of compounds 16, 19 and 20 in both systems (S2 and K4E7) is due to the absence of the >CH- group at the C6 position. This is confirmed both by the zero values of 2D descriptors *ES_Count_sssCH*, *ES_Sum_sssCH*, and by the contour maps of van der Waals interactions (Figs 7A and 9A). Also, it is quite obvious that compounds 17, 18 have low pIC_50_ values due to the presence of the -OH group in the C2 position instead of chlorine.

If we compare the 2D QSAR models for monoclonal antibodies (mAb) and polyclonal antibodies (pAb), one can see that both models (Model 4 for mAb and Model 7 for pAb) contain the polar surface area parameters *Jurs_FPSA_3* and *Jurs_TPSA*, respectively. The polar surface area is inversely correlated with the activity, which means that the polarity of atrazines is unfavorable for the interaction with both monoclonal and polyclonal antibodies. Both mAb and pAb are affected by the amount of methantriyl groups (*ES_Sum_sssCH* and *ES_Count_sssCH* parameters, respectively), which means that, in both cases, bulk substituents are favorable for higher activity. The latter statement is supported by 3D molecular interaction field contour maps (Figs 7A and 9A). We may see that both mAb and pAb systems have a great deal in common.

## Conclusions

In this study, we evaluated the efficiency of the interaction of 20 triazines with polyclonal and monoclonal antibodies grown using atrazine as the immunizing hapten.

The comparison of the two immunoassay systems showed that the system with the polyclonal antibodies (S2) is much easier to describe using 2D QSAR methodology, and the system with monoclonal antibodies can be described using the 3D QSAR CoMFA method. Thus, for the 3D QSAR model of the S2 system, the main statistical parameter q^2^ (LMO) is equal to 0.498, and for K4E7, q^2^ (LMO) = 0.566. Apparently, this is explained by the fact that in the case of polyclonal antibodies, we deal with several targets, while in interaction with monoclonal antibodies, the target is one, and therefore it is easier to describe it using specific fields of molecular interactions distributed in space.

There is an interesting fact: while for the S2 system the main contribution (72%) is made by steric interactions (S3 Table), for K4E7, the fraction of van der Waals and electrostatic interactions is equal (S4 Table).

Based on our analysis, we conclude that the effectiveness of the interaction with polyclonal antibodies significantly depends on the presence of the isopropylamino group in the C6 position of triazine, the presence of chlorine at the C2 position, and also the aniline substituent in the C4 position, whose substituents, in turn, participate in electrostatic interactions and hydrogen bonding.

The obtained data as well as the earlier-published results of QSAR technique application for immune recognition demonstrate the efficiency of this technique in the identification of key structures responsible for the distinguishing of structurally close antigens by antibodies. This information allows the theoretical formulation of criteria for molecules that could be recognized by the same antibodies (thus, extending the selectivity of one immunoassay) or requirements in different antibodies for detection (thus, realizing assays with mixtures or arrays of immunoreactants).

## Supporting information

S1 Fig Geometries of 20 triazine molecules aligned using compound 11 as a template. (PDF)

S1 Table The values of molecular descriptors from Model 5. (PDF)

S2 Table Values of molecular descriptors from Model 10. (PDF)

S3 Table Relative contribution (in %) of van der Waals forces and electrostatic interactions in 3D QSAR models for system S2. (PDF)

S4 Table Relative contribution (in %) of van der Waals forces and electrostatic interactions in the K4E7 model. (PDF)
