# Supplementary figures and images for: QSAR analysis of immune recognition for triazine herbicides based on immunoassay data for polyclonal and monoclonal antibodies
Source: PLoS One. 2019 Apr 3;14(4):e0214879. doi: 10.1371/journal.pone.0214879 (PMC6447172; doi:10.1371/journal.pone.0214879)

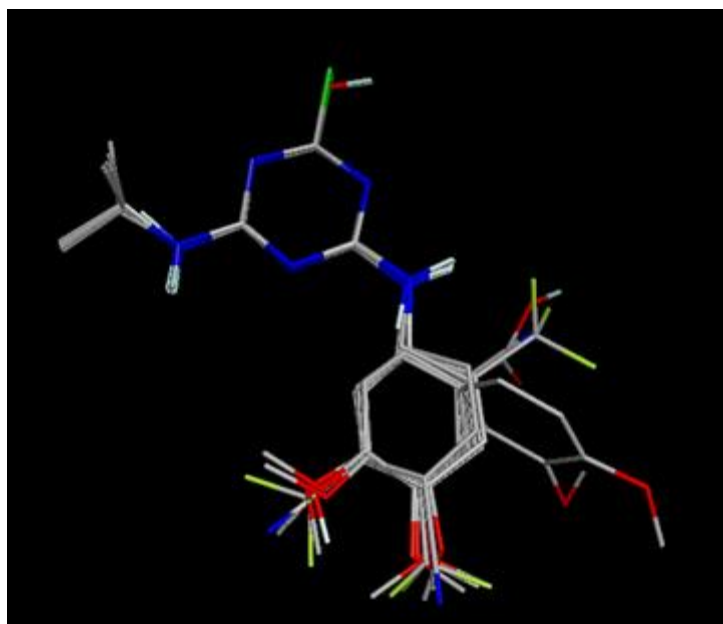

**Fig. S1.** Geometries of 20 triazine molecules aligned using compound **11** as a template.

Supplement: S1 Fig — (PDF) [file pone.0214879.s001.pdf]
