# Supplementary material for: QSAR analysis of immune recognition for triazine herbicides based on immunoassay data for polyclonal and monoclonal antibodies
Source: PLoS One. 2019 Apr 3;14(4):e0214879. doi: 10.1371/journal.pone.0214879 (PMC6447172; doi:10.1371/journal.pone.0214879)
Supplement: S1 Table — (PDF) [file pone.0214879.s002.pdf]

Table S1. The values of molecular descriptors from Model 5.

No	ES_Sum_sssCH	IC2	Solvation_E, kJ mol^{-1}
1	**0.266**	**3.431**	-33.49
2	0.238	3.482	-49.34
3	0	3.682	-56.07
4	0.214	3.529	-58
5	0.229	3.816	-34.75
6	0.187	4.178	-37.49
7	0.2	4.222	-43.14
8	0.167	4.425	-46.88
9	0.085	**4.426**	-43.43
10	0.173	4.15	-34.31
11	0.183	4.211	-42.64
12	0.19	4.09	-35.59
13	0.035	4.15	**-20.27**
14	0.069	4.207	-30.06
15	0.094	4.093	-27.35
16	0	4.263	-44.65
17	0.177	3.897	-62.89
18	0.134	4.241	**-71.22**
19	0	3.551	-52.42
20	0	3.978	-58.93
