# Supplementary material for: QSAR analysis of immune recognition for triazine herbicides based on immunoassay data for polyclonal and monoclonal antibodies
Source: PLoS One. 2019 Apr 3;14(4):e0214879. doi: 10.1371/journal.pone.0214879 (PMC6447172; doi:10.1371/journal.pone.0214879)
Supplement: S2 Table — (PDF) [file pone.0214879.s003.pdf]

**Table S2.**Values of molecular descriptors from Model 10.

| No | *ES_Count_sssCH* | *ES_Sum_sCl* | *Num_H_Donors_Lipinski* |
|---|---|---|---|
| 1 | 1 | 5.726 | 2 |
| 2 | 1 | 5.551 | 3 |
| 3 | 0 | 5.503 | 3 |
| 4 | 1 | 0 | 3 |
| 5 | 1 | 5.857 | 2 |
| 6 | 1 | 5.918 | 2 |
| 7 | 1 | 5.923 | 2 |
| 8 | 1 | 5.866 | 3 |
| 9 | 1 | 5.904 | 3 |
| 10 | 1 | 5.866 | 2 |
| 11 | 1 | 5.863 | 2 |
| 12 | 1 | 5.862 | 2 |
| 13 | 1 | 5.764 | 2 |
| 14 | 1 | 5.781 | 2 |
| 15 | 1 | 5.793 | 2 |
| 16 | 0 | 5.871 | 2 |
| 17 | 1 | 0 | 3 |
| 18 | 1 | 0 | 3 |
| 19 | 0 | 5.634 | 3 |
| 20 | 0 | 5.695 | 3 |
