# Supplementary material for: QSAR analysis of immune recognition for triazine herbicides based on immunoassay data for polyclonal and monoclonal antibodies
Source: PLoS One. 2019 Apr 3;14(4):e0214879. doi: 10.1371/journal.pone.0214879 (PMC6447172; doi:10.1371/journal.pone.0214879)
Supplement: S3 Table — (PDF) [file pone.0214879.s004.pdf]

Table S3. Relative contribution (in %) of van der Waals forces and electrostatic interactions in 3D QSAR models for system S2.

PC	van der Waals forces	electrostatic forces
1	60.88	39.12
2	72.48	27.52
3	72.31	27.69
