# Supplementary material for: QSAR analysis of immune recognition for triazine herbicides based on immunoassay data for polyclonal and monoclonal antibodies
Source: PLoS One. 2019 Apr 3;14(4):e0214879. doi: 10.1371/journal.pone.0214879 (PMC6447172; doi:10.1371/journal.pone.0214879)
Supplement: S4 Table — (PDF) [file pone.0214879.s005.pdf]

Table S4. Relative contribution (in %) of van der Waals forces and electrostatic interactions in the K4E7 model.

PC	van der Waals forces	electrostatic forces
1	51.9	48.1
2	52.7	47.3
3	51.2	48.8
